# Medical Leave Associated With COVID-19 Among Emergency Medical System Responders and Firefighters in New York City

**DOI:** 10.1001/jamanetworkopen.2020.16094

**Published:** 2020-07-24

**Authors:** David J. Prezant, Rachel Zeig-Owens, Theresa Schwartz, Yang Liu, Karen Hurwitz, Shenecia Beecher, Michael D. Weiden

**Affiliations:** 1 The Bureau of Health Services and the FDNY World Trade Center Health Program, Fire Department of the City of New York, Brooklyn, New York; 2 Office of Medical Affairs, Fire Department of the City of New York, Brooklyn, New York; 3 Pulmonary Medicine Division, Department of Medicine, Montefiore Medical Center and Albert Einstein College of Medicine, Bronx, New York; 4 Division of Epidemiology, Department of Epidemiology and Population Health, Albert Einstein College of Medicine, Bronx, New York; 5 Pulmonary, Critical Care and Sleep Medicine Division, Department of Medicine and Department of Environmental Medicine, New York University School of Medicine, New York, New York

## Abstract

This cohort study examines use of medical leave among emergency medical service (EMS) responders and firefighters in New York, New York, during the coronavirus disease 2019 (COVID-19) pandemic compared with earlier periods.

## Introduction

In New York, New York, from March 1 to May 31, 2020, 201 102 individuals were diagnosed with coronavirus disease 2019 (COVID-19), resulting in 51 085 hospitalizations and 16 834 deaths.^1^ The Fire Department of the City of New York (FDNY), the largest in the US, responds to nearly 1.5 million emergency medical calls per year in a city of more than 8.4 million people. Active paid FDNY responders include 4408 emergency medical service (EMS) responders and 11 230 firefighters. These FDNY responders are required to don personal protective equipment before patient contact per US Centers for Disease Control and Prevention guidelines.^2^ In this cohort study, we compared medical leave of FDNY responders during the pandemic with prior years.

## Methods

The Montefiore Medical Center/Albert Einstein College of Medicine institutional review board deemed this study was exempt from review and informed consent owing to minimal risk to participants (ie, no effect on their rights and welfare). This study followed the Strengthening the Reporting of Observational Studies in Epidemiology (STROBE) reporting guideline.

Paid medical leave is provided for FDNY responders. Medical leave dates and diagnoses (including COVID-19) were obtained from the FDNY electronic medical record from October 1, 2017, to May 31, 2020. Suspected COVID-19 was based on history, symptoms, and, when available, chest radiographic imaging. Diagnosis of COVID-19 was confirmed with reverse transcriptase–polymerase chain reaction (RT-PCR) testing. Medical leave for COVID-19 was managed via telemedicine. Return-to-duty decisions followed Centers for Disease Control and Prevention guidelines for health care personnel.^3^ Descriptive analyses in the form of counts and means, depending on data type, were conducted. The incidence of COVID-19–related medical leave was calculated between March 1 and May 31, 2020. Person-time accrual began on March 1 2020, and ended on date of COVID-19 diagnosis for individuals with confirmed COVID-19 and May 31, 2020, for individuals without confirmed COVID-19. For Poisson distribution, 95% CIs were calculated. Analyses were performed using SAS statistical software version 9.4 (SAS Institute).

## Results

As of March 31, 2020, 1792 of 4408 EMS responders (40.7%) and 3873 of 11 230 firefighters (34.5%) were on medical leave for suspected (3894 individuals [68.7%], including 1219 EMS responders and 2675 firefighters) or confirmed (1771 individuals [31.3%], including 573 EMS responders and 1198 firefighters) COVID-19, among whom 66 (1.2%) were hospitalized and 4 died. The COVID-19 incidence rate was 4.21 (95% CI, 4.02-4.41) per 100 person-weeks for EMS responders and 3.2 (95% CI, 3.19-3.39) per 100 person-weeks for firefighters. The mean (SD) age was 35.8 (10.2) years for EMS responders and 38.9 (8.3) years for firefighters. Among EMS responders, 1305 (72.8%) were men, and among firefighters, 3830 (98.9%) were men. FDNY responders with COVID-19 diagnoses were similar in age and sex to the whole workforce.

**Figure 1.**
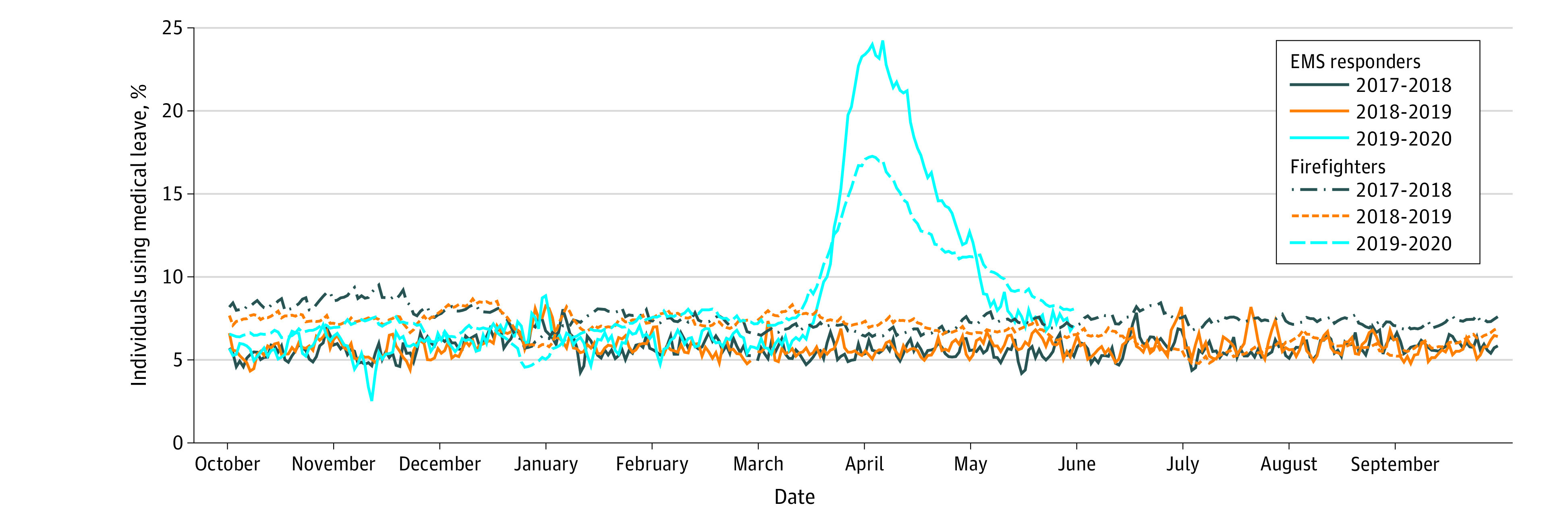
The Proportion of Fire Department of the City of New York First Responders on Medical Leave From October 1, 2017, to May 31, 2020, During the COVID-19 Pandemic and Previous Influenza Periods COVID-19 indicates coronavirus disease 2019; EMS, emergency medical services.

**Figure 2.**
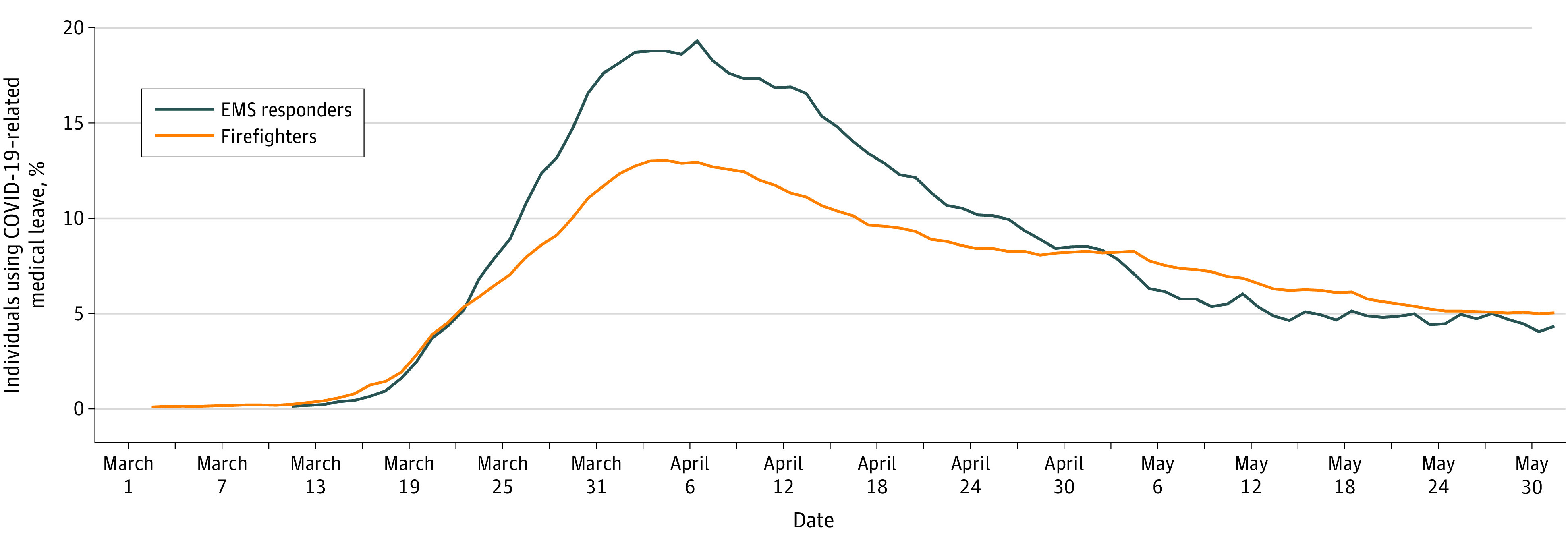
The Proportion of Fire Department of the City of New York First Responders on Medical Leave Associated With COVID-19 Diagnosis From March 1 to May 31, 2020 COVID-19 indicates coronavirus disease 2019; EMS, emergency medical services.

In March 2020, medical leave for EMS responders and firefighters increased above baseline (EMS responders: 6.1%; firefights: 6.8%), even when compared with months during influenza periods in prior years, with the greatest number on medical leave in early April (Figure 1). COVID-19 diagnosis fully accounted for this increase, reaching its peak of 19.3% for EMS responders and 13.0% for firefighters (Figure 2). Physicians and nurses placed nearly 400 calls per day to FDNY responders on medical leave—calling every 2 to 3 days to assess fever and symptoms and daily to those with dyspnea or worsening symptoms. By March 31, 2020, 1391 EMS responders and 3098 firefighters had returned to duty, reducing COVID-19 medical leave to 4.3% among EMS responders and 5.0% among firefighters. Among those who returned to duty, the mean (SD) medical leave duration was 25.3 (13.2) days among 1469 individuals with COVID-19 confirmed by RT-PCR testing and 19.8 (12.0) days among 3020 individuals with suspected COVID-19 that was not confirmed.

## Discussion

Among FDNY EMS responders and firefighters, the COVID-19 pandemic was associated with increased medical leave, reducing the workforce available to meet COVID-19–associated EMS surge demands in New York. Medical leave among FDNY responders increased rapidly, peaking the first week of April. The higher rate of COVID-19 illness in EMS responders vs firefighters likely reflects greater exposure to patients with COVID-19 during delivery of prehospital medical care. This occurred despite providing identical personal protective equipment to all FDNY responders. Increased COVID-19 infection rates among health care workers has been reported at 1.1% by RT-PCR confirmation in China^4^ and 14% to 18% by clinical history in the Netherlands.^5^

Effective management of medical leave was essential to maintaining the integrity of the EMS system during this pandemic, and clinical follow-up is ongoing. Limitations of this study include combining suspected and confirmed COVID-19 diagnosis owing to limited availability of diagnostic testing; the inability to determine whether personal protective equipment was used appropriately^6^; and lack of data on what portion of medical leave resulted from community rather than work-related exposures. By including data from the entire FDNY cohort and comparing them with data from prior years, the potential for ascertainment and selection biases were minimized. These results could prove useful to other cities facing rapid escalation of COVID-19 infections.
