# Error in Results Section

**DOI:** 10.1001/jamanetworkopen.2020.19975

**Published:** 2020-08-20

In the Original Investigation titled “Medical Leave Associated With COVID-19 Among Emergency Medical System Responders and Firefighters in New York City,”^1^ published July 24, 2020, there were errors in the dates given in the first sentence of the first paragraph and the second-to-last sentence of the final paragraph. Both should have appeared as May 31, 2020, rather than March 31, 2020. This article has been corrected.^1^
